# Histamine and Histamine H4 Receptor Promotes Osteoclastogenesis in Rheumatoid Arthritis

**DOI:** 10.1038/s41598-017-01101-y

**Published:** 2017-04-26

**Authors:** Kyoung-Woon Kim, Bo-Mi Kim, Kyung-Ann Lee, Sang-Heon Lee, Gary S. Firestein, Hae-Rim Kim

**Affiliations:** 1 Convergent Research Consortium in Immunologic Disease, Seoul St. Mary’s Hospital, College of Medicine, The Catholic University of Korea, Seoul, Korea; 2 Department of Rheumatology, Research Institute of Medical Science, Konkuk University School of Medicine, Seoul, Korea; 3 Division of Rheumatology, Allergy and Immunology, University of California San Diego, La Jolla, California United States of America

## Abstract

Histamine H4 receptor (H4R) has immune-modulatory and chemotaxic effects in various immune cells. This study aimed to determine the osteoclastogenic role of H4R in rheumatoid arthritis (RA). The concentration of histamine in synovial fluid (SF) and sera in patients with RA was measured using ELISA. After RA SF and peripheral blood (PB) CD14+ monocytes were treated with histamine, IL-17, IL-21 and IL-22, and a H4R antagonist (JNJ7777120), the gene expression H4R and RANKL was determined by real-time PCR. Osteoclastogenesis was assessed by counting TRAP–positive multinucleated cells in PB CD14+ monocytes cultured with histamine, Th17 cytokines and JNJ7777120. SF and serum concentration of histamine was higher in RA, compared with osteoarthritis and healthy controls. The expression of H4R was increased in PB monocytes in RA patients. Histamine, IL-6, IL-17, IL-21 and IL-22 induced the expression of H4R in monocytes. Histamine, IL-17, and IL-22 stimulated RANKL expression in RA monocytes and JNJ7777120 reduced the RANKL expression. Histamine and Th17 cytokines induced the osteoclast differentiation from monocytes and JNJ7777120 decreased the osteoclastogenesis. H4R mediates RANKL expression and osteoclast differentiation induced by histamine and Th17 cytokines. The blockage of H4R could be a new therapeutic modality for prevention of bone destruction in RA.

## Introduction

Rheumatoid arthritis (RA) is a systemic autoimmune disorder, characterized by chronic synovitis of peripheral joints, subsequent cartilage and bone destruction followed by joint disability^1, 2^. Various cellular receptors and intracellular signal molecules have been highlighted in RA pathogenesis because the inhibition of their biologic function showed significant improvement of clinical symptoms and signs in RA patients^3–7^.

Histamine is a short-acting biological amine that is widely distributed throughout the human body^8–10^. The role of histamine in the pathogenesis of RA inflammation is largely unknown. A few studies determining histamine has pro-inflammatory or anti-inflammatory role in RA is still controversial. Histamine is present in RA blood, synovial tissue, synovial fluid (SF) and articular cartilage^11–14^. While T cells, macrophages and neutrophils synthesize synovial histamine, the main cells producing histamine are mast cells. Mast cells are increased in RA synovial tissues and cartilage-pannus junctions^15^. In RA, chondrocytes produce histamine which stimulates the production of prostaglandin E and matrix metalloproteinase (MMP)-1, causing joint damages^16–19^. These results suggest histamine is pro-inflammatory molecule in RA pathogenesis. On the contrary, the injection of histamine into mouse knee joints induces no signs of synovitis^20^. Serum and SF levels of histamine are lower in RA patients than in healthy volunteers and the histamine level increases with controlling RA disease activity with tumor necrosis factor (TNF)-α inhibitor^20^. The clinical and laboratory studies for the role of histamine in RA do not coincide, but the discordance has not been deduced. Although the role of histamine in RA pathogenesis is obscure, in the basis of the pathogenesis and pathology, histamine could be proinflammatory and tissue destructive molecules in RA.

The biological function of histamine is mediated through 4 different histamine receptors, histamine H1~H4 receptors^9, 10, 21^. They have different unique characteristics in their expression, function, and signal transduction^10, 22^. Histamine H4 receptor (H4R) is a G-protein coupled receptor and it is expressed mainly on various immune cells such as mast cells, eosinophils, monocytes, dendritic cells, T cells and natural killer cells^23^. Previous studies suggest H4R has a key role in inflammatory process of RA. H4R is expressed in RA synovial tissues, vessels and synovial cells, both macrophage-like and fibroblast-like synoviocytes^24–27^. In K/BxN serum transfer mice, a H4R antagonist, clozapine, protects mice from arthritis symptoms and histological cartilage and bone damage^28^. In collagen-induced arthritis and collagen antibody-induced arthritis models, H4R deficiency and H4R blockage ameliorate clinical arthritis, histological damage and number of Th17 cells^29, 30^. The mechanisms that H4R antagonist controls arthritis are reduction of pro-inflammatory cytokines and mediators, nuclear factor NF-κB, MMP-3, and a pro-inflammatory receptor, glucocorticoid-induced TNFR-related protein, while H4R antagonist increases number of blood CD4+ CD25+ FOXP3+ Treg cells, and the expression of interleukin (IL)-10 and transforming growth factor-β^29, 30^. However, one study shows the gene expression of H4R is decreased in RA synovial tissue compared with osteoarthritis, it has negative correlation with MMP-3 and serum levels of anti-cyclic citrullinated peptide antibody and C-reactive protein^31^. So, the role of H4R in RA pathogenesis needs to be clarified.

This study aimed to determine the role of histamine and H4R in osteoclastogenesis of RA. We examined the role of histamine on induction of the receptor activator of nuclear factor-κB ligand (RANKL) and osteoclast differentiation from its precursors. We also determined the mediation of H4R in the histamine and Th17 cytokine-induced RANKL expression and osteoclastogenesis. Therefore, we suggest the blockage of H4R could be a new therapeutic modality for prevention of bone destruction in RA patients.

## Results

### SF and serum concentration of histamine in RA patients

**Figure 1:**
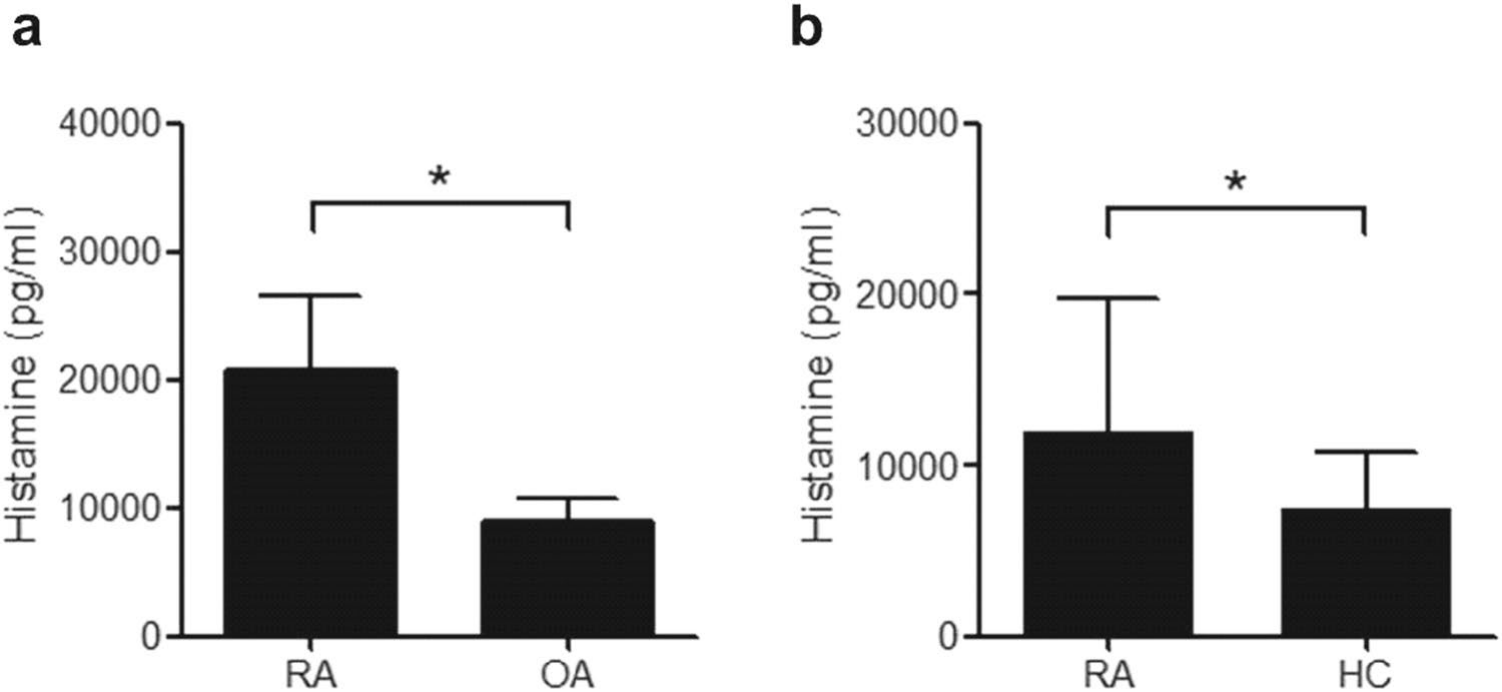
The synovial fluid (SF) and serum concentration of histamine in RA patients. (**a**) The SF concentration of histamine in RA and osteoarthritis patients was measured using ELISA. (**b**) The serum concentration of histamine in RA patients and healthy controls was measured using ELISA. The data represent the mean ± SEM of three independent experiments. *P < 0.05. RA; rheumatoid arthritis, OA; osteoarthritis, HC; healthy control.

The SF and serum concentration of histamine was measured using ELISA kit in 40 patients with RA (18 patients for SF and 19 patients for serum), 20 patients of osteoarthritis and 19 healthy controls. The clinical characteristics for RA patients who provided their SF were as follows; 3 males and 15 females, age 57.1 ± 3.9 years, ESR 50.2 ± 8.6 mm/h, CRP 3.3 ± 0.7 mg/dl, and rheumatoid factor 65 ± 20.0 IU/ml. The SF concentration of histamine was higher in RA patients than in osteoarthritis patients (20,639 ± 5,824 pg/ml vs 8,872 ± 1,799 pg/ml, *P* < 0.05, Fig. 1a). The clinical characteristics for RA patients who provided their sera were as follows; 4 males and 15 females, age 55.3 ± 4.0 years, ESR 55.9 ± 8.7 mm/h, CRP 3.4 ± 0.8 mg/dl, and rheumatoid factor 111.4 ± 27.7 IU/ml. The serum concentration of histamine was also higher in RA patients than in healthy controls (11,894 ± 7,786 pg/ml vs 7,490 ± 3,279 pg/ml, *P* < 0.05, Fig. 1b). There was no correlation between the histamine concentration in SF and sera with the clinical measures (data not shown).

### Basal expression of H4R mRNA in SF and PB CD14+ monocytes of RA patients

**Figure 2:**
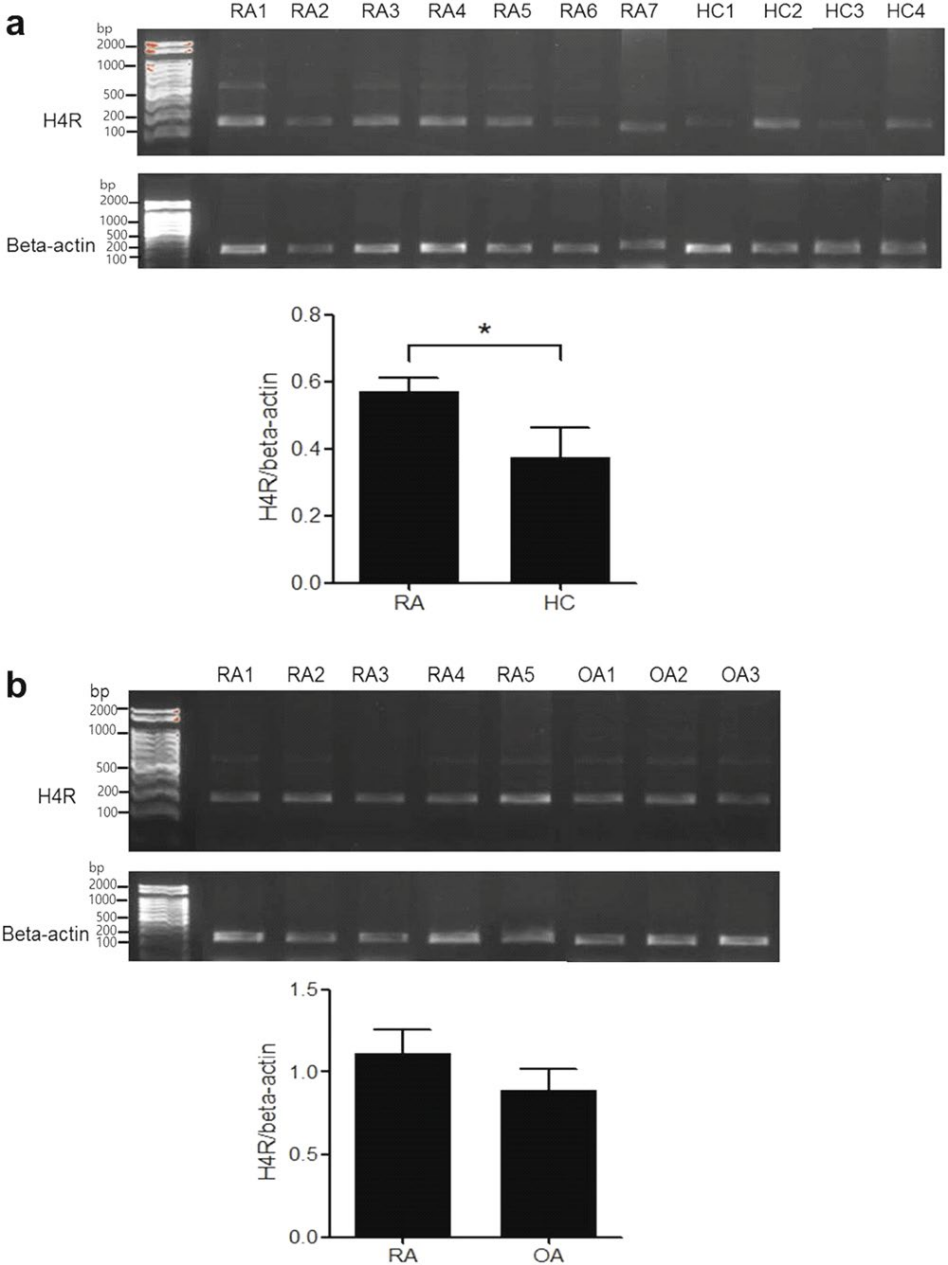
The expression of histamine H4 receptor (H4R) in peripheral blood (PB) and synovial fluid (SF) monocytes of patients with rheumatoid arthritis (RA). (**a**) The basal expression of H4R mRNA in PB CD14+ monocytes from 7 RA patients and 4 healthy controls. (**b**) The basal expression of H4R mRNA in SF monocytes from 5 RA patients and 3 osteoarthritis patients. Data were normalized to beta-actin and reported in relative expression units. The data represent the mean ± SEM of three independent experiments. *P < 0.05. H4R; histamine H4 receptor, RA; rheumatoid arthritis, OA; osteoarthritis, HC; healthy control.

The basal expression of H4R mRNA in PB monocytes was increased in RA patients, compared with healthy controls (*P* < 0.05, Fig. 2a). However, there was no statistical difference of the H4R mRNA expression in SF monocytes between RA and OA patients (Fig. 2b).

### The expression of H4R mRNA induced by histamine and inflammatory cytokines in CD14+ monocytes

**Figure 3:**
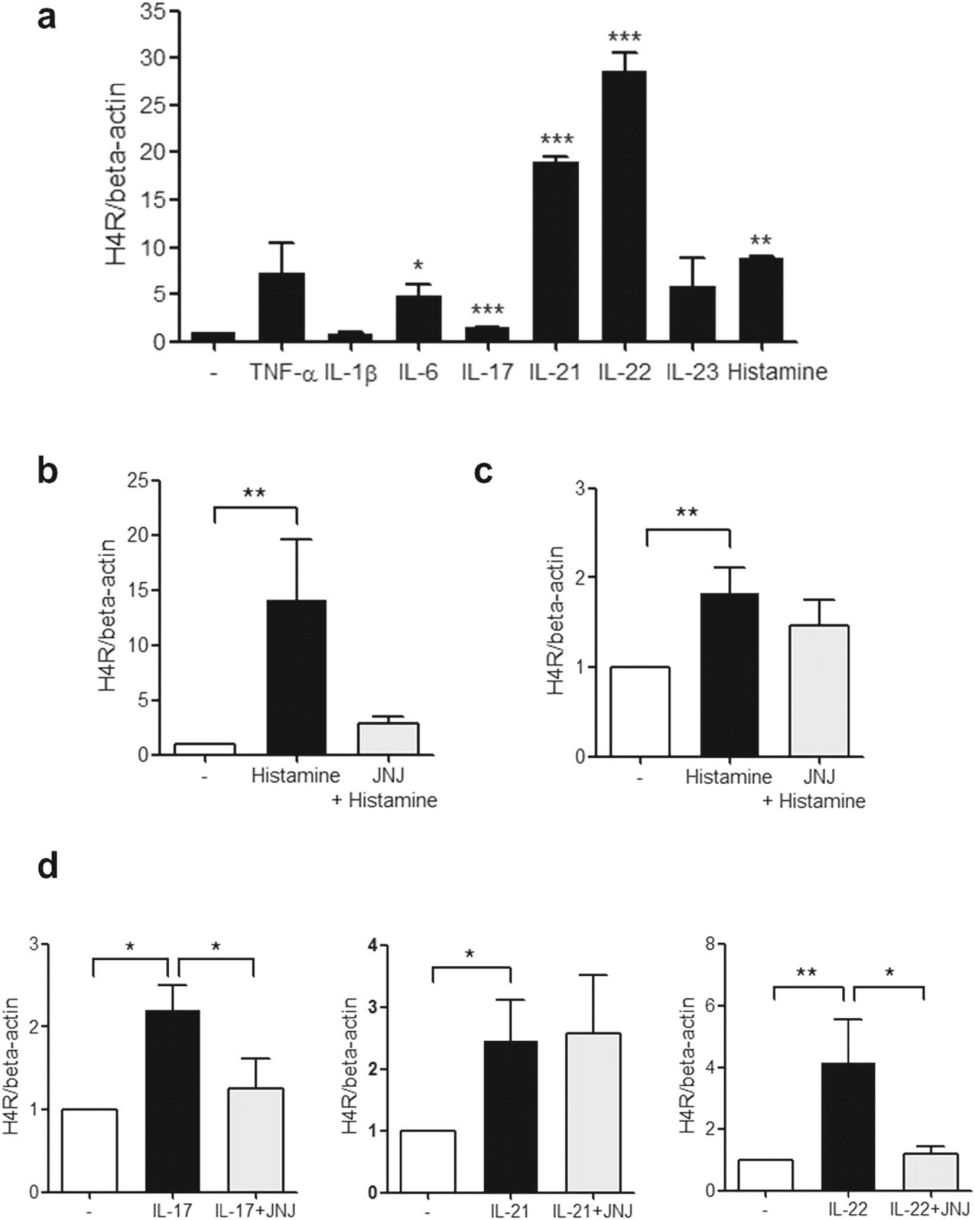
The expression of H4R mRNA induced by histamine and inflammatory cytokines. (**a**) After PB monocytes were cultured with 20 ng/ml of TNF-α, IL-1β, IL-6, IL-17, IL-21, IL-22, IL-23 and 10^−5^ M of histamine for 16 h, the expression of H4R mRNA was determined by real-time PCR. (**b**) After PB CD14+ monocytes from 3 RA patients were cultured with 10^−5^ M of histamine and 10 μM of JNJ7777120, a selective H4R antagonist, for 16 h, the expression of H4R mRNA was determined by real-time PCR. (**c**) After SF CD14+ monocytes from 3 RA patients were cultured with 10^−5^ M of histamine and 10 μM of JNJ7777120 for 16 h, the expression of H4R mRNA was determined by real-time PCR. (**d**) After PB monocytes from 3 RA patients were cultured with IL-17, IL-21, IL-22 and 10 μM of JNJ7777120 for 16 h, the expression of H4R mRNA was determined by real-time PCR. Data were normalized to beta-actin and reported in relative expression units. The data represent the mean ± SEM of three independent experiments. *P < 0.05, **P < 0.01 and ***P < 0.001. H4R; histamine H4 receptor, JNJ; JNJ7777120.

To determine the effective cytokines for induction of H4R expression, CD14+ monocytes from PB of RA patients were cultured with histamine and various inflammatory cytokines such as TNF-α, IL-1β, IL-6, IL-17, IL-21, IL-22 and IL-23. Histamine, IL-6, IL-17, IL-21 and IL-22 significantly increased the expression of H4R (Fig. 3a) in PB monocytes. Especially, the stimulatory effect of IL-21 and IL-22 on the H4R expression was much larger than histamine, so we selected the Th17 cytokines, IL-17, IL-21 and IL-22, as the interesting cytokines for further studies. When CD14+ monocytes from PB of RA patients and healthy controls and cultured with various doses of histamine (0–10^−4^ M), the H4R mRNA expression was increased with maximal effect of 10^−5^ M of histamine (data not shown). In PB and SF monocytes of RA patients, the histamine stimulated H4R expression and JNJ7777120, a selective H4R antagonist, tended to reduce the histamine stimulation, however, there was no statistical significance (Fig. 3b,c). IL-17, IL-21 and IL-22 increased H4R expression in PB monocytes of RA patients and JNJ7777120 reversed IL-17 and IL-22-induced H4R expression (Fig. 3d).

### The expression of RANKL mRNA induced by histamine and Th17 cytokines in CD14+ monocytes

**Figure 4:**
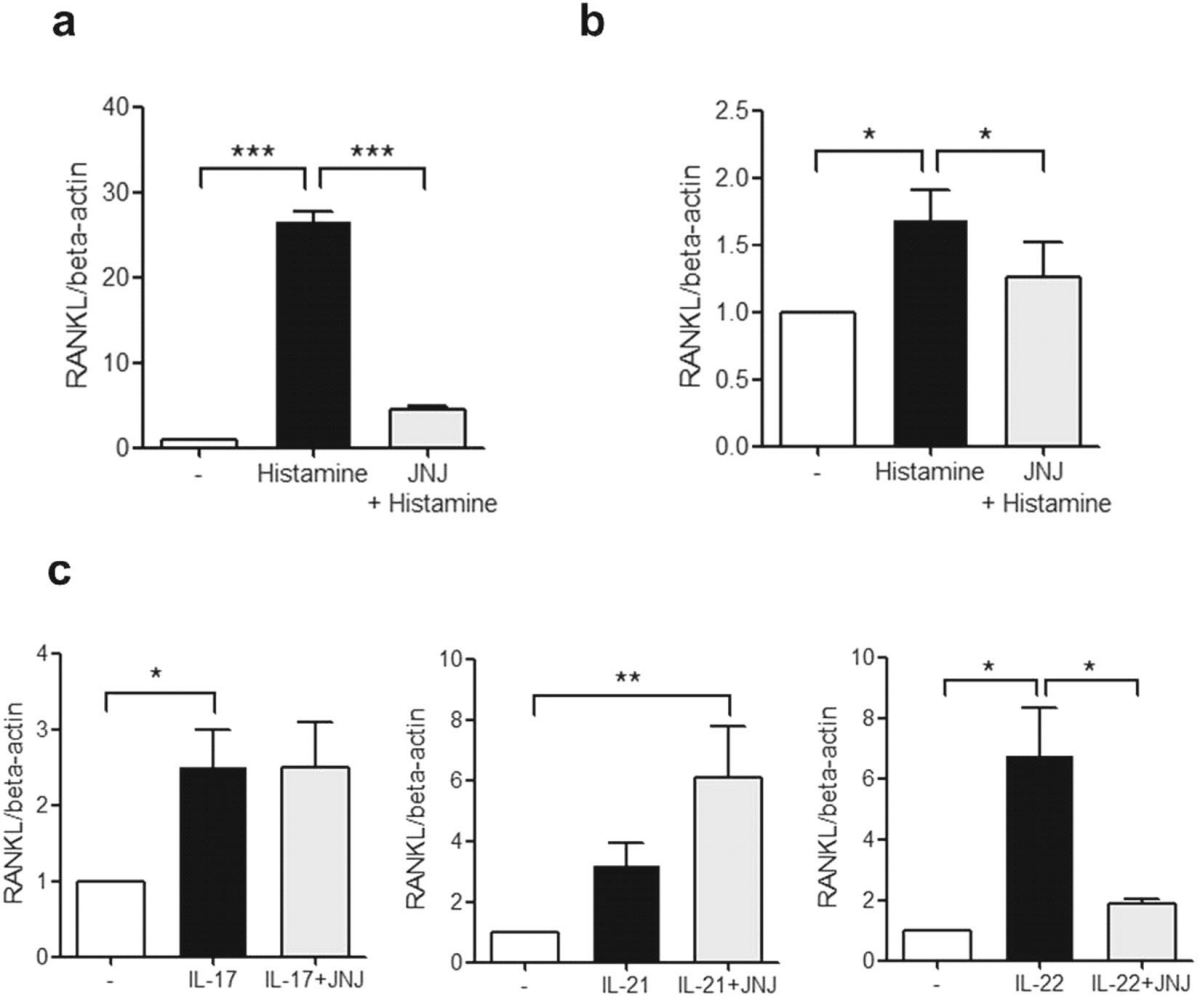
The expression of RANKL mRNA induced by histamine and Th17 cytokines. (**a**) After PB monocytes from 3 RA patients were cultured with 10^−5^ M of histamine and 10 μM of JNJ7777120 for 16 h, the expression of RANKL mRNA was determined by real-time PCR. (**b**) After SF monocytes from 3 RA patients were cultured with 10^−5^ M of histamine and 10 μM of JNJ7777120 for 16 h, the expression of RANKL mRNA was determined by real-time PCR. (**c**) After PB monocytes from 3 RA patients were cultured with IL-17, IL-21, IL-22 and 10 μM of JNJ7777120 for 16 h, the expression of RANKL mRNA was determined by real-time PCR. Data were normalized to beta-actin and reported in relative expression units. The data represent the mean ± SEM of three independent experiments. *P < 0.05, and ***P < 0.001. H4R; histamine H4 receptor, JNJ; JNJ7777120.

The expression of RANKL mRNA of CD14+ monocytes of PB and SF from RA patients was increased by histamine and it decreased by JNJ7777120 (Fig. 4a,b). IL-17 and IL-22 stimulated the expression of RANKL in PB monocytes and JNJ7777120 reduced the IL-22-induced RANKL expression (Fig. 4c). However, IL-21 did not increase RANKL expression.

### Histamine-induced osteoclast differentiation in PB monocytes

**Figure 5:**
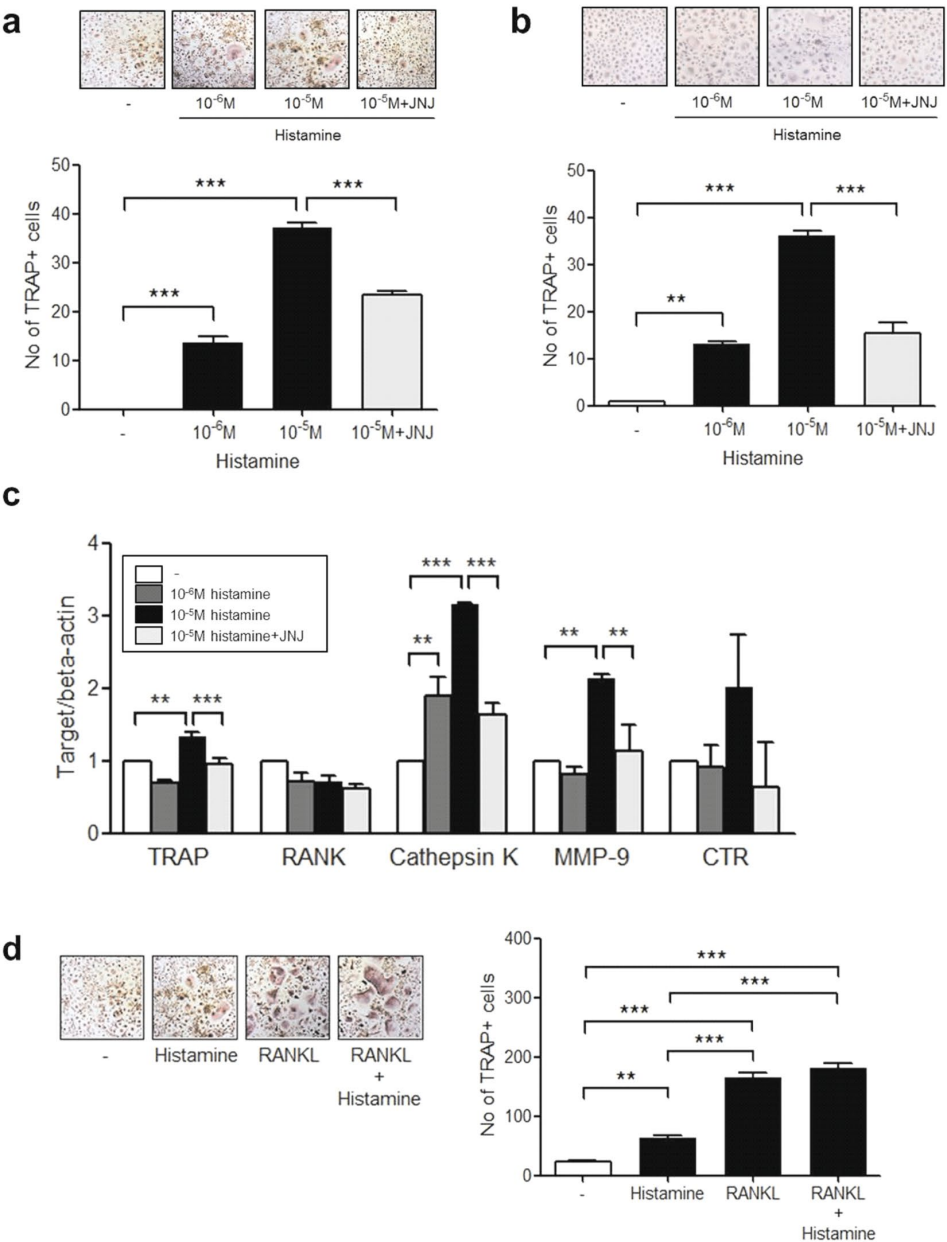
Histamine-induced osteoclast differentiation in PB monocytes and the inhibitory effect of H4R antagonist in RANKL-induced osteoclastogenesis. (**a**) CD14+ monocytes isolated from PB of HC were cultured with 0–10^−5^ M of histamine and 10 μM of JNJ7777120 in the presence of 25 ng/ml of M-CSF. After 21 days of culturing, TRAP-positive multinucleated cells were counted. The figures represent one of 3 independent experiments. (**b**) CD14+ monocytes isolated from PB of 3 RA patients were cultured with 0–10^−5^ M of histamine and 10 μM of JNJ7777120 in the presence of 25 ng/ml of M-CSF. After 21 days of culturing, TRAP-positive multinucleated cells were counted. The figures represent one of three independent experiments. (**c**) CD14+ monocytes were cultured with 0–10^−5^ M of histamine and 10 μM of JNJ7777120 in the presence of 25 ng/ml M-CSF and the mRNA expression of osteoclast markers such as TRAP, RANK, cathepsin K, MMP-9 and CTR was determined using real-time PCR. (**d**) CD14+ monocytes were cultured with 10 ng/ml of RANKL, 10^−5^ M histamine, and RANKL with histamine in the presence of 25 ng/ml of M-CSF. After 21 days of culturing, TRAP-positive multinucleated cells were counted. The figures represent one of three independent experiments. The figures represent one of three independent experiments. The data represent the mean ± SEM of three independent experiments. **P < 0.01 and ***P < 0.001. TRAP; tartrate-resistant acid phosphatase, JNJ; JNJ7777120, RANK; receptor activator of nuclear factor-κB, MMP-9; matrix metalloproteinase-9, CTR; calcitonin receptor.

PB CD14+ monocytes can differentiate into osteoclasts when they are cultured with RANKL and M-CSF. To determine the osteoclastogenic effect of histamine, CD14+ monocytes isolated from PB were cultured with histamine and M-CSF in the absence of RANKL. After 21 days of culture, TRAP+ multinucleated osteoclasts were differentiated in the culture with histamine and JNJ7777120 reduced the histamine-induced osteoclastogenesis in osteoclast precursors from RA patients and healthy controls (Fig. 5a,b). The gene expression of osteoclast markers, such as TRAP, cathepsin K, and MMP-9 was significantly increased with histamine stimulation and decreased with JNJ7777120 (Fig. 5c). There was no synergistic or additive effect of histamine and RANKL in osteoclast differentiation (Fig. 5d).

### The mediation of H4R in Th17 cytokine-induced osteoclast differentiation

**Figure 6:**
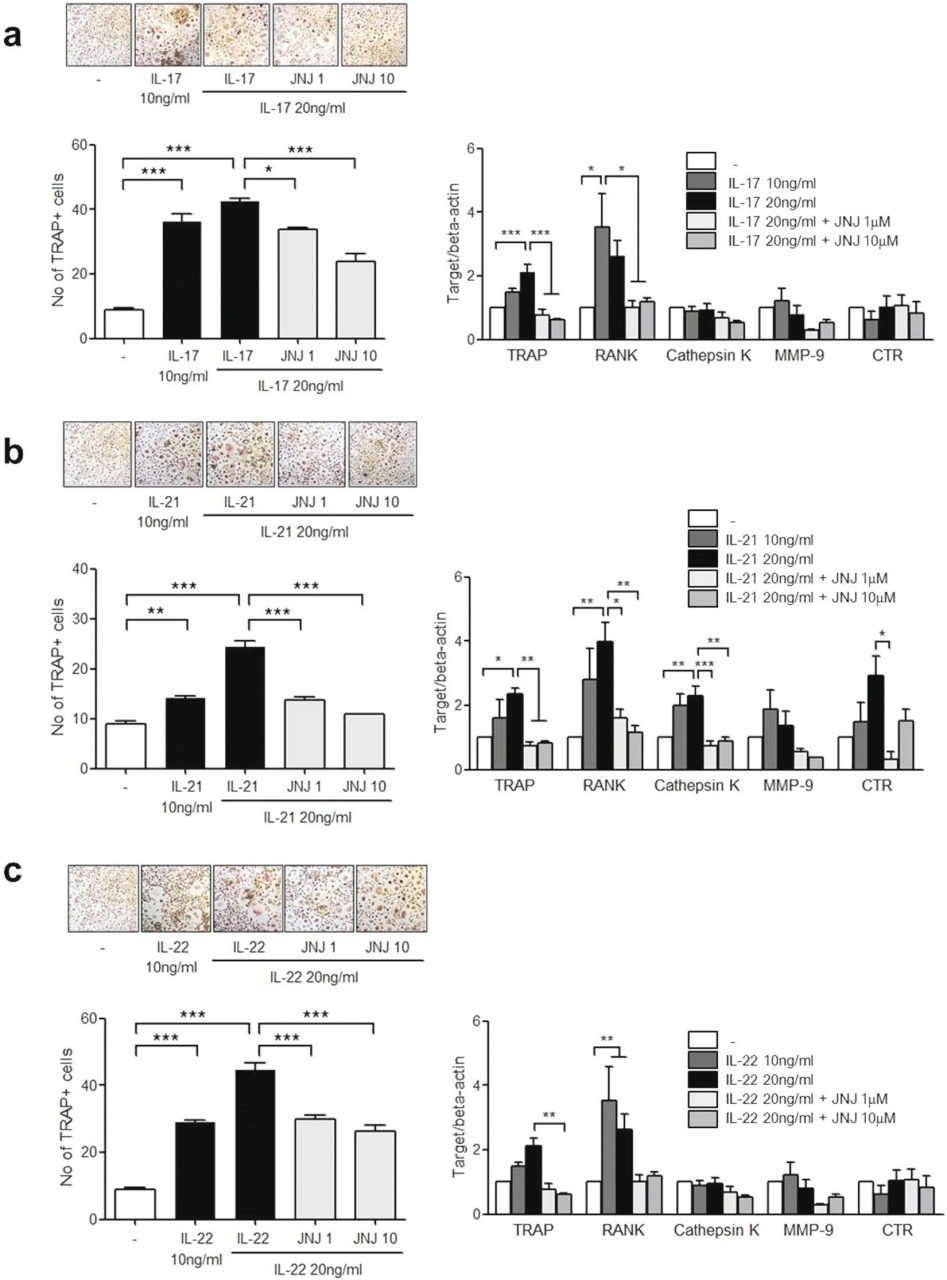
Th17 cytokine-induced osteoclast differentiation in monocytes and the effect of H4R antagonist. (**a**) PB monocytes isolated were cultured with 10–20 ng/ml of IL-17 and 1–10 μM of JNJ7777120 in the presence of 25 ng/ml of M-CSF. After 21 days of culturing, TRAP-positive multinucleated cells were counted and the mRNA expression of osteoclast markers such as TRAP, RANK, cathepsin K, MMP-9 and CTR was determined using real-time PCR. (**b**) PB monocytes were cultured with 10–20 ng/ml of IL-21 and 1–10 μM of JNJ7777120 in the presence of 25 ng/ml of M-CSF. After 21 days of culturing, TRAP-positive multinucleated cells were counted and the mRNA expression of osteoclast markers such as TRAP, RANK, cathepsin K, MMP-9 and CTR was determined using real-time PCR. (**c**) PB monocytes were cultured with 10–20 ng/ml of IL-22 and 1–10 μM of JNJ7777120 in the presence of 25 ng/ml of M-CSF. After 21 days of culturing, TRAP-positive multinucleated cells were counted and the mRNA expression of osteoclast markers such as TRAP, RANK, cathepsin K, MMP-9 and CTR was determined using real-time PCR. The figures represent one of three independent experiments. The data represent the mean ± SEM of three independent experiments. *P < 0.05, **P < 0.01 and ***P < 0.001. TRAP; tartrate-resistant acid phosphatase, JNJ; JNJ7777120, RANK; receptor activator of nuclear factor-κB, MMP-9; matrix metalloproteinase-9, CTR; calcitonin receptor.

PB CD14+ monocytes were cultured with IL-17, IL-21 or IL-22 in the presence of M-CSF. After 21 days of culture, TRAP+ multinucleated osteoclasts were differentiated with the stimulation of the three cytokines in a dose dependent manner. JNJ7777120 reduced the Th17 cytokine-induced osteoclastogenesis in a dose dependent manner (*P* < 0.001, Fig. 6a–c).

## Discussion

Although H4R is characterized by its unique role in inflammatory, autoimmune and allergic diseases, there are still not clear studies for determination of its role in RA pathogenesis^32^. In this study, we aimed to define the role of histamine and its receptor, H4R on the osteoclastogenesis in RA. H4R has a major role in controlling inflammation and joint destruction in preclinical animal models^28–30^ and mast cells and histamine is present in pannus-bone junction and they increase monocyte recruitment close to bone surface^15, 33^. On the basis of these results, histamine and its receptors could involve in bone destructive process of RA. However, there has not been *in vitro* study for their osteoclastogenic roles using biologic samples from RA patients. We hypothesized histamine could have a role in osteoclastogenesis when it is mediated thorough H4R and the osteoclastogenic effect of Th17 cytokines could be mediated through H4R in RA.

For determination of clinical significance of histamine in RA, we measured the histamine concentration in RA SF and serum. In RA, SF and serum concentration of histamine was increased, compared with controls. In previous studies, there is much lower concentration of histamine in RA SF and sera^20, 34, 35^. The inconsistency between the studies and our study, it is possible that they measured it with different methods such as fluorometric method or radioimmunoassay, while we measured histamine concentration using ELISA kit. There are controversial results among the studies for the clinical meaning of histamine in RA. When RA disease activity is divided three groups as DAS28, very active group has the highest histamine index (SF histamine concentration/serum histamine concentration×100) and inactive group has the lowest histamine index^34^. However, another study shows serum concentration of histamine has negative correlation with CRP and DAS28 score, and after treating with infliximab, the serum histamine concentration increases^20^.

To examine the basal expression of H4R in RA cells, we compared the expression of H4R mRNA in PB and SF monocytes between patients with RA, OA and healthy controls. The expression of H4R mRNA was significantly higher in RA PB monocytes than in OA PB monocytes, while there was no difference in the expression of H4R mRNA between RA and OA SF monocytes. H4R is mainly expressed in bone marrow-originated cells such as mast cells, eosinophils, monocytes, and T cells. Our preliminary study showed that PB, but not SF, mononuclear cells expressed higher H4R in RA patients than in health controls (data not shown). This result supports blood cells express much higher H4R than synovial cells, however, in RA synovial unit, macrophage-like synoviocytes, fibroblast-like synoviocytes, chondrocytes and osteoclasts express H4R^27^. There is lower expression of H4R in RA synovial tissues than in OA synovial tissues and the authors explain negative feedback effect of the expression of H4R by repeated stimulation of the receptor^31^. However, because the concentration of histamine is lower in RA SF^20^, it is possible that H4R could have systemic effect rather than localized effect in the joints, mediating by other inflammatory molecules, not by histamine.

Previous study shows histamine promotes osteoclastogenesis through H1R and H2R, however, there has been no study for the role of histamine and H4R on osteoclastogenesis^33^. The major mechanism of osteoclastognesis in RA synovium is the upregulation of RANKL in synovial and immune cells and the activation of synovial osteoclast precursors^36, 37^. In our study, histamine up-regulated RANKL and H4R expression in RA PB and SF monocytes, and this effect was inhibited by JNJ7777120, a selective H4R antagonist. This result suggests histamine induces RANKL expression in both peripheral circulation and local joints, which has a potential for activation of osteoclast precursors and H4R mediates the histamine-induced RANKL expression. Although the pattern of histamine-induced RANKL expression was similar in PB monocytes of healthy volunteers and RA patients and SF monocytes of RA patients, there was the strongest effect of histamine-induced RANKL expression PB monocytes from healthy volunteers. Because histamine level is lower in RA patients^20, 38^ and circulating and synovial mononuclear cells of RA patients are already stimulated by many inflammatory stimuli, histamine and its immunologic response could be down-regulated in RA local synovial environment.

Histamine induced osteoclast differentiation from its potential precursors, CD14+ monocytes, in the presence of M-CSF. There are two possible mechanisms in histamine-induced osteoclastogenesis. First, histamine directly induced osteoclastogenesis, independent of RANKL stimulation. Second, histamine stimulated the monocytes and other synovial cells to express RANKL, and then they were differentiated into mature osteoclasts with RANKL produced by autocrine and paracrine effects. There is controversy in the role of histamine in autoimmune diseases, it is not confirmed whether it has anti-inflammatory or pro-inflammatory role. In this study, histamine has definite osteoclastogenic role through RANKL induction and direct induction of osteoclast differentiation, mediating by H4R.

As well as histamine, IL-6 and Th17 family cytokines, IL-17, IL-21 and IL-22, stimulated H4R expression. Especially IL-21 and IL-22 had stronger effect for H4R stimulation than histamine. The Th17 cytokines strongly induced RANKL expression and this effect was mediated through H4R. This result is consistent with the *in vivo* study that treatment with H4R antagonist reduces the number of IL-17 positive cells in the lymph nodes^29^. It is already known that IL-17, IL-21 and IL-22 have osteoclastogenic role in RA pathogenesis^39, 40^. In our recent study, they stimulate RANKL expression and directly induce osteoclast differentiation and synovial fibroblasts which are exposed by Th17 cytokines also induces osteoclastogenesis^41^. H4R is directly stimulated by Th17 cytokines and H4R antagonist reduced the Th17 cytokine-induced osteoclast differentiation. This result suggests Th17 cytokine-induced H4R expression could be another mechanism for osteoclastogenesis induced by these cytokines. As well as their unique receptors, Th17 cytokines could augment the osteoclastogenesis via additional stimulation for another inflammatory receptor, H4R. Among the Th17 cytokines, IL-17 and IL-22 had strong effect for the induction of H4R and RANKL expression and the differentiation into osteoclasts among three cytokines, so IL-22 or IL-17/H4R axis could be potential therapeutic target for RA-associated bone destruction. Because this study did not investigate the role of IL-6 in H4R-mediated osteoclastogenesis, IL-6 would be further investigated in this area.

There was phase II clinical study of toreforant, a novel, orally active, selective H4R antagonist, in patients with active RA despite MTX therapy. Although toreforant 100 mg/day improved DAS28-CRP and ACR20 response in 12 weeks, the phase IIa study terminated prematurely because of fatal adverse effect of hemophagocytic lymphohistiocytosis. In phase IIb study reduced the dose of toreforant because of the fatal adverse effect. In the reduced doses, there was no significant improvement in DAS28-CRP in week 12^42^. Insufficient doses with short duration showed no significant result, so circumstantial study for the relationship of the drug and adverse effect should indicate appropriate doses and study duration. Moreover, other H4R antagonists with different antagonistic mechanisms would be further developed.

Histamine concentration and H4R expression in PB monocytes were increased in RA patients. H4R could play a major role in bone destruction of RA, by mediating RANKL expression and osteoclast differentiations which is induced by histamine and Th17 cytokines. The inhibition of the linkage between H4R and Th17 cytokines could be a new therapeutic option for preventing bone destruction in RA.

## Methods

### Ethics Statement and Patients

Written informed consent was obtained from patients with RA and osteoarthritis and healthy volunteers. All RA patients fulfilled the 1987 revised criteria of the American College of Rheumatology (ACR)^43^. SF was obtained from 3 RA patients (age 56.7 ± 12.7 years, 1 male and 2 females, disease duration 6.8 ± 1.1 years, erythrocyte sedimentation rate (ESR) 46.7 ± 36.2 mm/h, C-reactive protein (CRP) 3.5 ± 3.3 mg/dl) and 3 patients with knee osteoarthritis (age 70.3 ± 1.3 year, all females). All SF was obtained by therapeutic arthrocentesis before steroid injection into swollen joints. Peripheral blood (PB) was obtained from 3 RA patients (age 53.7 ± 4.8 years, disease duration 1.1 ± 0.5 years, ESR 47.0 ± 18.3 mm/h, CRP 0.7 ± 0.7 mg/dl) and healthy volunteers. All methods were performed in accordance with the relevant guidelines and regulations. Written informed consent was obtained from patients with RA and osteoarthritis and healthy volunteers. The protocol for this study was approved by Institutional Review Board for Human Research in Konkuk University Hospital.

### Reagents

Recombinant TNF-α, IL-1β, IL-6, IL-17, IL-21, IL-22 and IL-23 were purchased from R&D systems (Minneapolis, MN, USA). Histamine and a H4R antagonist, JNJ7777120, were purchased from the Sigma Chemical Co. (St Louis, MA, USA).

### ELISA for measuring SF and serum concentration of histamine

SF was obtained from patients with RA and osteoarthritis. Serum was obtained from patients with RA and healthy volunteers. SF and serum were measured for histamine content by Histamin ELISA kit according to the manufacturer’s instructions (Enzo Life Sciences Inc., NY, USA).

### Expression of H4R mRNA by reverse transcription–polymerase chain reaction (RT-PCR)

PB and SF monocytes were incubated with various concentrations of histamine and cytokines in the presence or absence of JNJ7777120. After 16 hours of incubation, mRNA was extracted with RNAzol B (Biotex Laboratories, Houston, TX, USA) in accordance with the manufacturer’s instructions. Reverse transcription of 2 μg of total mRNA was performed at 42 °C using the Superscript™ reverse transcription system (Takara, Shiga, Japan). PCR amplification of cDNA aliquots was performed by adding 2.5 mM dNTPs, 2.5 U of *Taq* DNA polymerase (Takara) and 0.25 μM of sense and antisense primers. The reaction was performed in PCR buffer (1.5 mM MgCl2, 50 mM KCl, 10 mM Tris-HCl, pH 8.3) in a total volume of 25 μl. Reactions were processed in a DNA thermal cycler (Perkin- Elmer Cetus, Norwalk, CT, USA) through cycles for 30 seconds of denaturation at 94 °C, 1 min of annealing at 60 °C (β-actin) or 59 °C (*H4r*), followed by 1 min of elongation at 72 °C. PCR rounds were repeated for 25 cycles each for *H4r* and *beta-actin*; this was determined as falling within the exponential phase of amplification for each molecule. The level of mRNA expression was presented as a ratio of Target PCR product over *beta-actin* product.

### Real-time PCR for quantitation of H4R and RANKL mRNA

mRNA was extracted using RNAzol B according to the instructions of the manufacturer (Biotex). Reverse transcription of total mRNA (2 μg) was performed at 42 °C using a SuperScript RT system (Takara). PCR was performed in a final volume of 20 μl in capillary tubes in a LightCycler (Roche Diagnostics). The reaction mixture contained 2 μl LightCycler FastStart DNAMaster Mix for SYBR Green I (Roche Diagnostics), 0.5 μ*M* of each primer, 4 m*M* MgCl2, and 2 μl of template DNA. All of the capillaries were amplified in a LightCycler instrument with activation of polymerase (95 °C for 10 minute), followed by 45 cycles of 10 seconds at 95 °C, 10 seconds at 60 °C (*beta-actin*) or 59 °C (*H4r*, *Rankl*, *Trap*, *Rank*, *Ctr*, *Mmp-9*), and 10 seconds at 72 °C. The temperature transition rate was 20 °C/second for all steps. The following sense and antisense primers for each molecules were used: *H4r* sense, 5′-GGA AGC GTG ATC ATC TCA GTA GGT-3′; *H4r* antisense, 5′-TCT GAA TGA GTG TCC ACG ATG TT-3′; *Rankl* sense, 5′-ACC AGC ATC AAA ATC CCA AG-3′; *Rankl* antisense, 5′-CCC CAA AGT ATG TTG CAT CC-3′; *Trap* sense, 5′-GAC CAC CTT GGC AAT GTC TCT G-3′; *Trap* antisense, 5′-TGG CTG AGG AAG TCA TCT GAG TTG-3′; *Rank* sense, 5′-GCT CTA ACA AAT GTG AAC CAG GA-3′; *Rank* antisense, 5′-GCC TTG CCT GTA TCA CAA ACT-3′; *Cathepsin K* sense, 5′-TGA GGC TTC TCT TGG TGT CCA TAC-3′; *Cathepsin K* antisense, 5′-AAA GGG TGT CAT TAC TGC GGG-3′; *Mmp-9* sense, 5′-CGC AGA CAT CGT CAT CCA GT-3′; *Mmp-9* antisense, 5′-GGA TTG GCC TTG GAA GAT GA-3′; *Calcitonin receptor* sense, 5′-TGG TGC CAA CCA CTA TCC ATG C-3′; *Calcitonin receptor* antisense, 5′-CAC AAG TGC CGC CAT GAC AG-3′; *beta-actin* sense, 5′-GGA CTT CGA GCA AGA GAT GG-3′; *beta-actin* antisense, 5′-TGT GTT GGC GAT CAG GTC TTT- G-3′. Melting curve analysis was performed immediately after the amplification protocol, under the following conditions: 0 seconds (hold time) at 95 °C, 15 seconds at 71 °C, and 0 seconds (hold time) at 95 °C. The rate of temperature change was 20 °C/second except in the final step, during which it was 0.1 °C/second. The melting peak generated represented the quantity of specific amplified product. The crossing point was defined as the maximum of the second derivative from the fluorescence curve. Negative controls that contained all elements of the reaction mixture except for the template DNA were also included. All samples were processed in duplicate.

### Monocyte isolation and osteoclast differentiation

PBMCs and SFMCs were separated and washed three times with sterile PBS and resuspended in RPMI 1640 (Life Technologies, Grand Island, NY, USA) supplemented with 10% fetal bovine serum (FBS), 2 mM L-glutamine, and 1% penicillin-streptomycin, henceforth called complete medium. Freshly isolated PBMCs and SFMCs were incubated at 37 °C in complete medium and allowed to adhere for 45 minutes. The non-adherent cells were removed and the adherent cells were washed with sterile PBS, harvested with a rubber policeman, and stained with the monocyte-specific anti-CD14 monoclonal antibody to assess the purity of the preparation. Ninety percent of the isolated cells were monocytes expressing CD14. The osteoclast precursors were prepared using the monocytes-enriched fraction from PB. The cells were co-cultured for 3 weeks in minimal essential medium (MEM)-α and 10% heat-inactivated FBS in the presence of 25 ng/ml rhM-CSF and histamine/Th17 cytokines/JNJ7777120. The medium was changed on day 3 and then every other day thereafter. On day 21, tartrate-resistant acid phosphatase (TRAP)-positive cells were identified using a leukocyte acid phosphatase kit according to the manufacturer’s protocol (Sigma-Aldrich).

### Statistical analysis

Results are expressed as mean ± standard error of the mean (SEM). Statistical differences were assessed using Mann-Whitney *U* test for analyzing two groups or one-way analysis of variance (ANOVA) with Bonferroni’s multiple comparison post-hoc test for analyzing more than three groups. A *P* value < 0.05 was considered statistically significant.
